# Supplementary material for: Integrated view and comparative analysis of baseline protein expression in mouse and rat tissues
Source: PLoS Comput Biol. 2022 Jun 17;18(6):e1010174. doi: 10.1371/journal.pcbi.1010174 (PMC9246241; doi:10.1371/journal.pcbi.1010174)
Supplement: S4 File — Supplementary figures (A) illustrating correlation of protein abundances in organs represented in different datasets. (B) Correlation of protein abundances generated using iBAQ and spectral counting methods in various mouse organs. (C) Correlation of protein expression between organs within human, mouse and rat. (PDF) [file pcbi.1010174.s004.pdf]

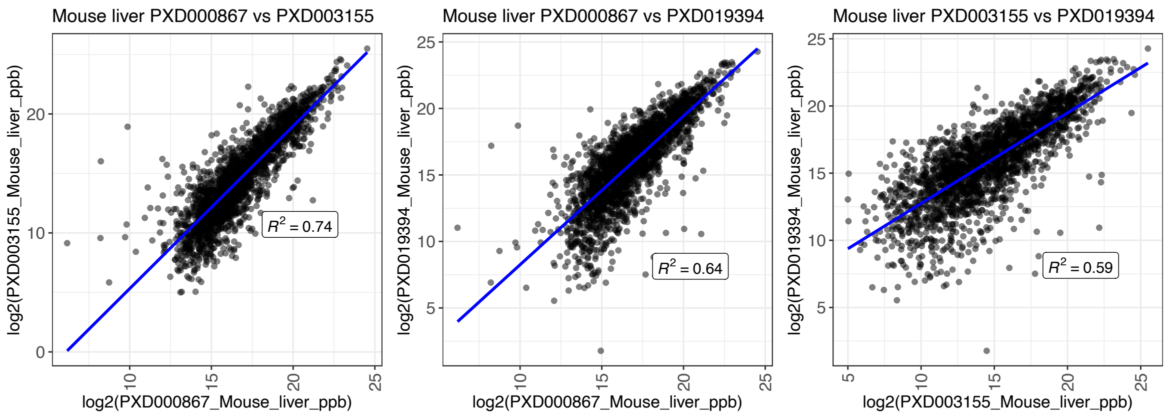

Supplementary Figure A. Correlation of protein expression between datasets for mouse liver.

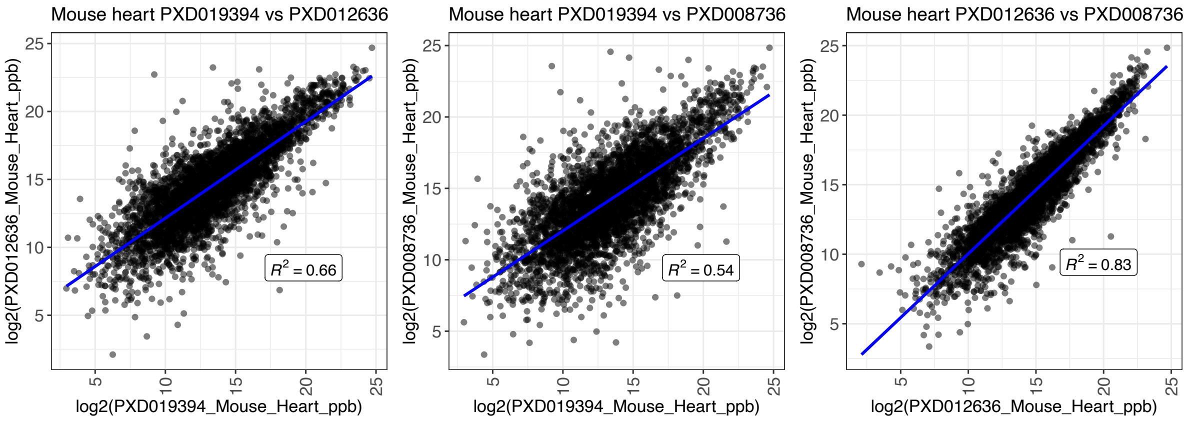

Supplementary Figure B. Correlation of protein expression between datasets for mouse heart.

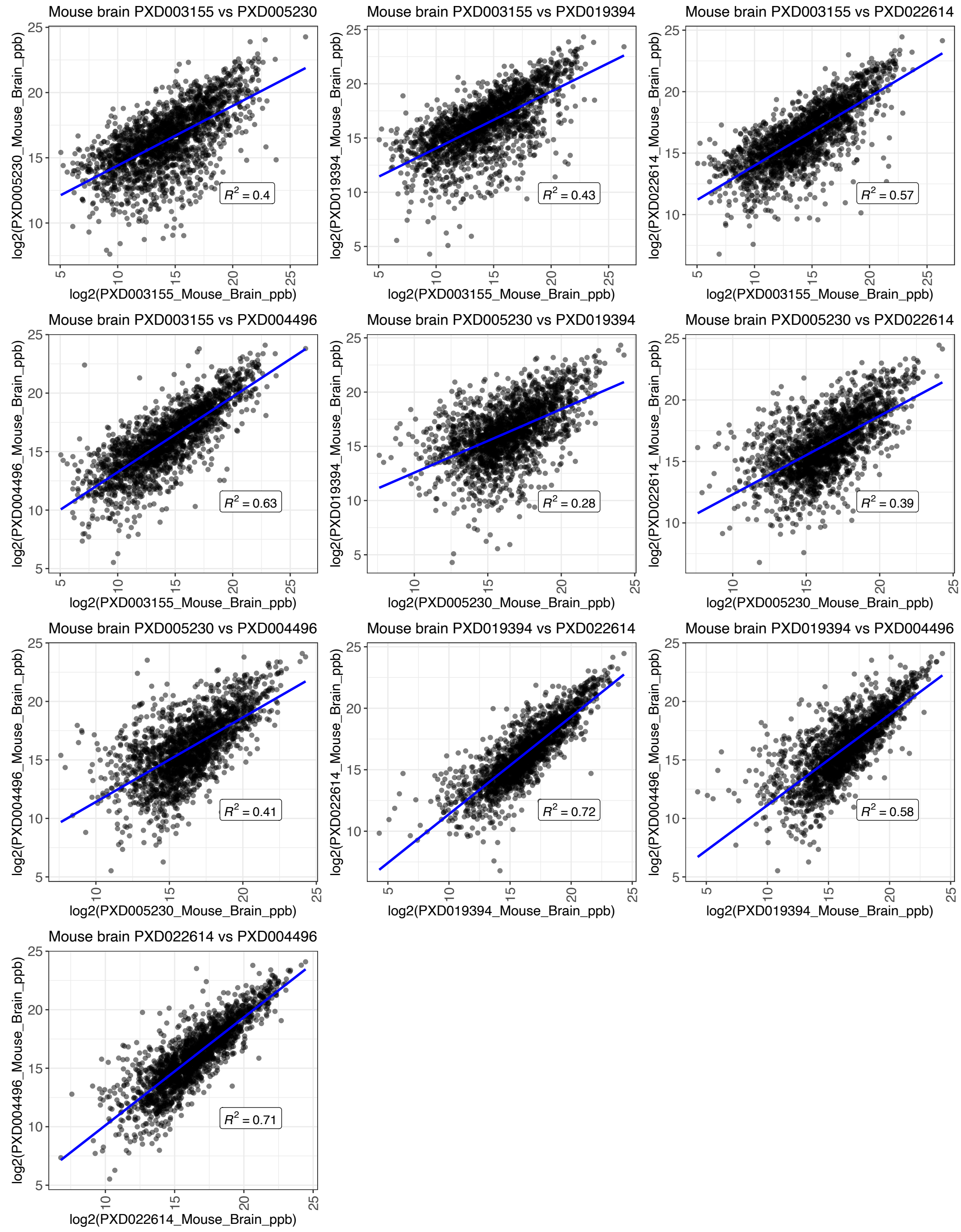

Supplementary Figure C. Correlation of protein expression between datasets for mouse brain.

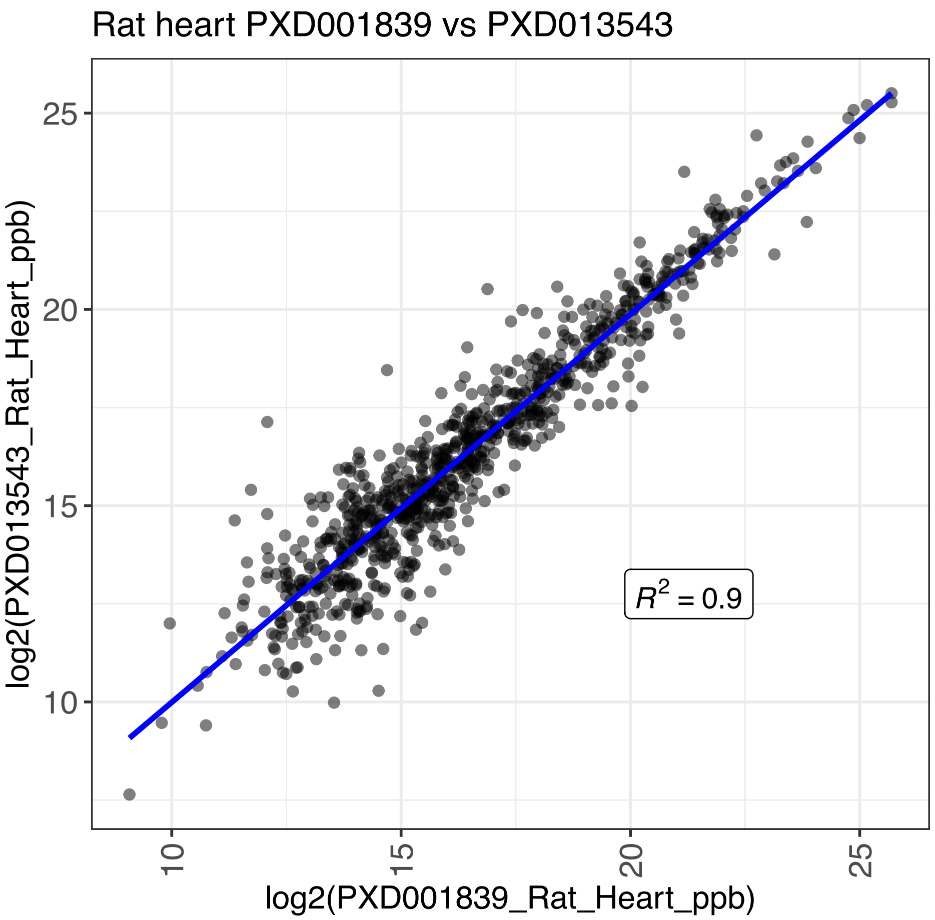

Supplementary Figure D. Correlation of protein expression between datasets for rat heart.

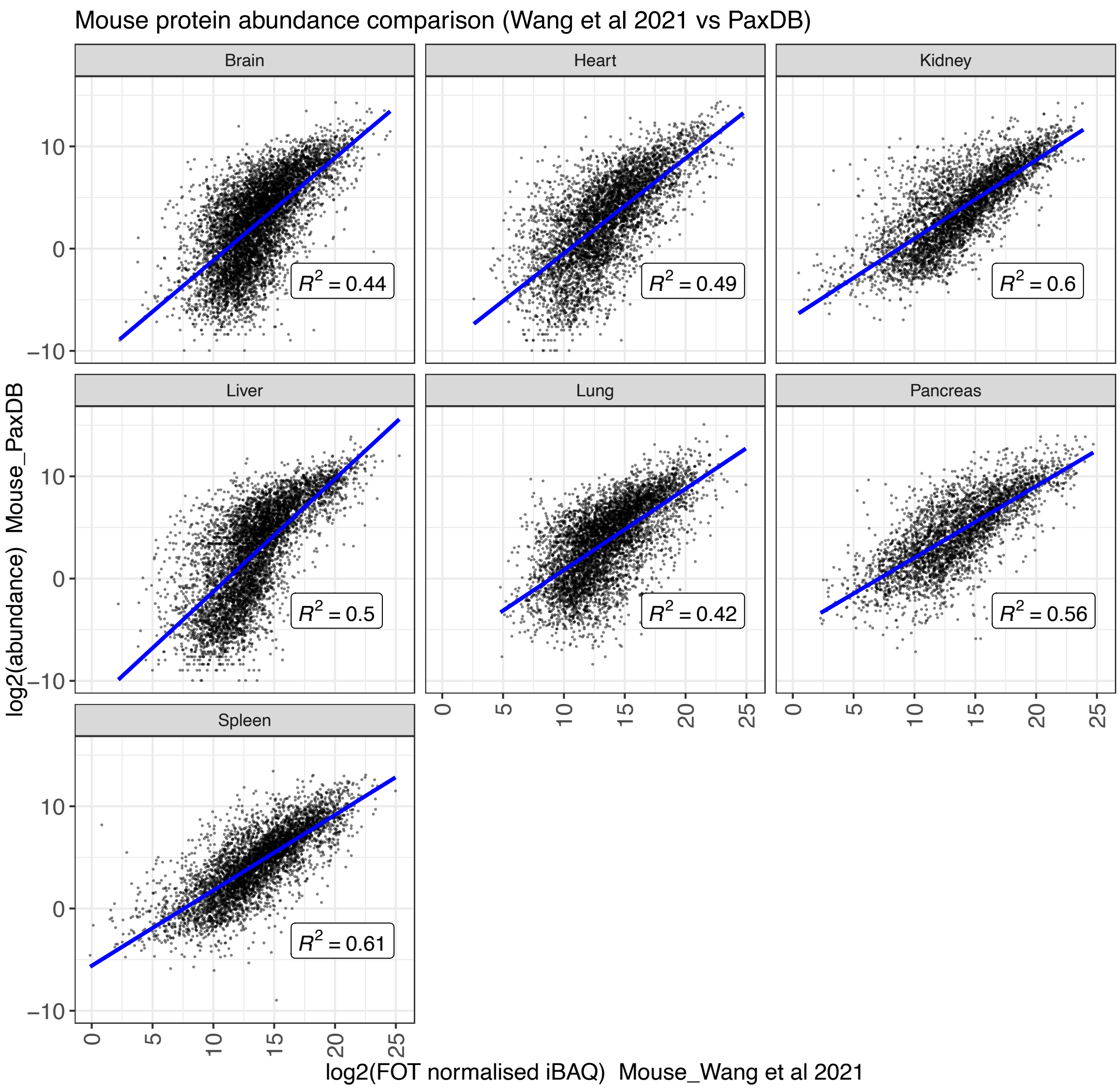

Supplementary Figure E. Comparison of mouse protein abundances in various organs between this study (iBAQ) and PaxDB (spectral counting).

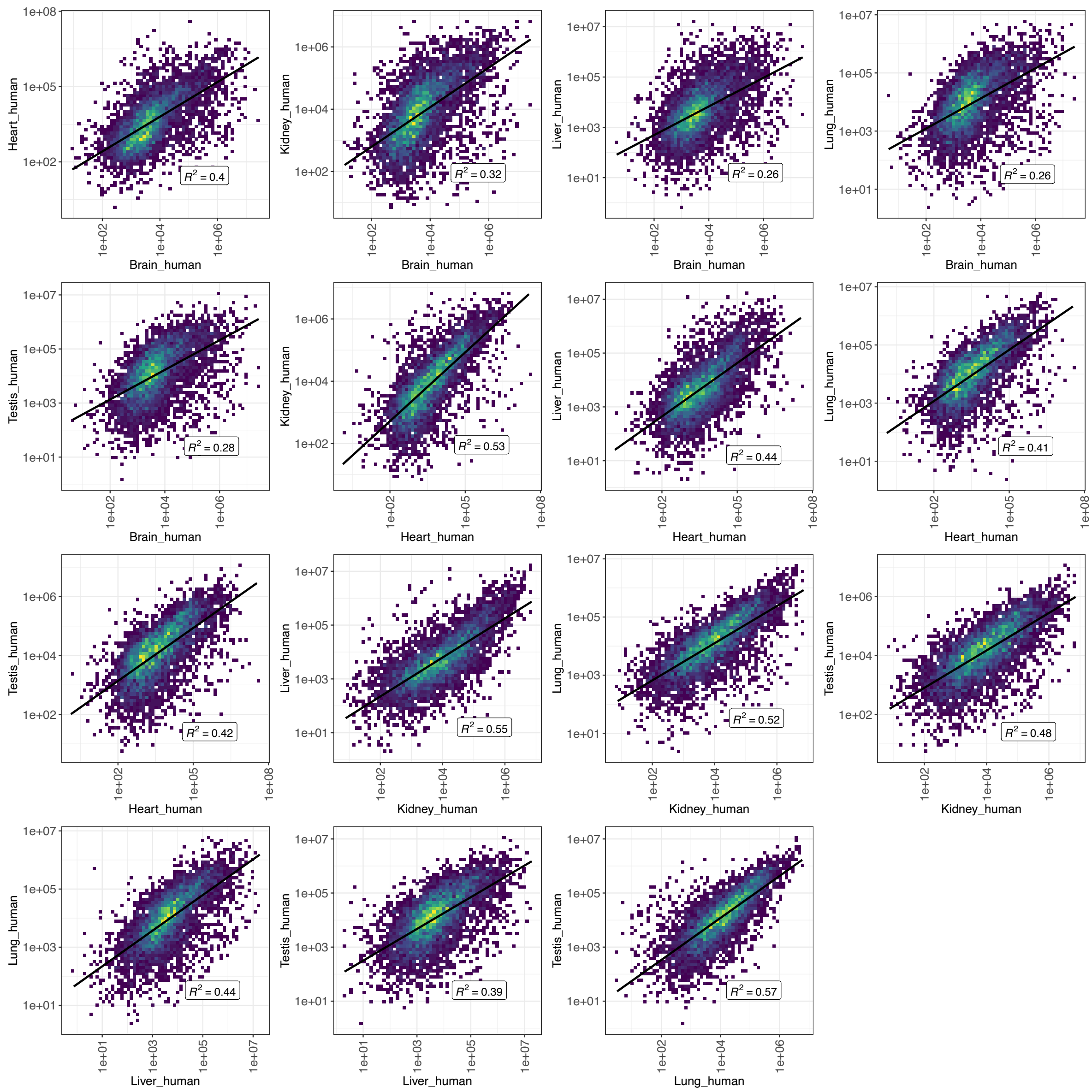

Supplementary Figure F. Correlation of protein expression between organs within human.

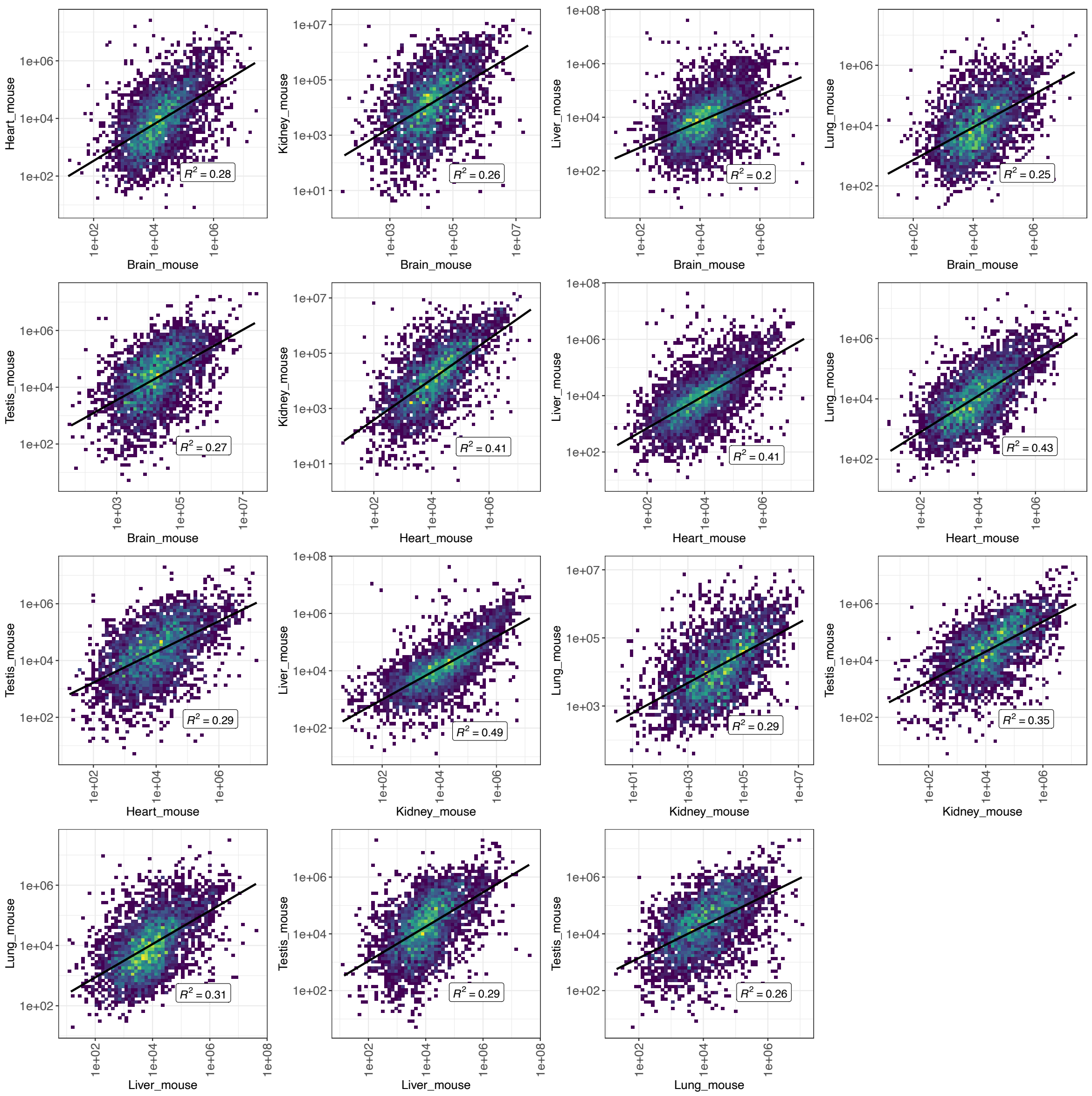

Supplementary Figure G. Correlation of protein expression between organs within mouse.

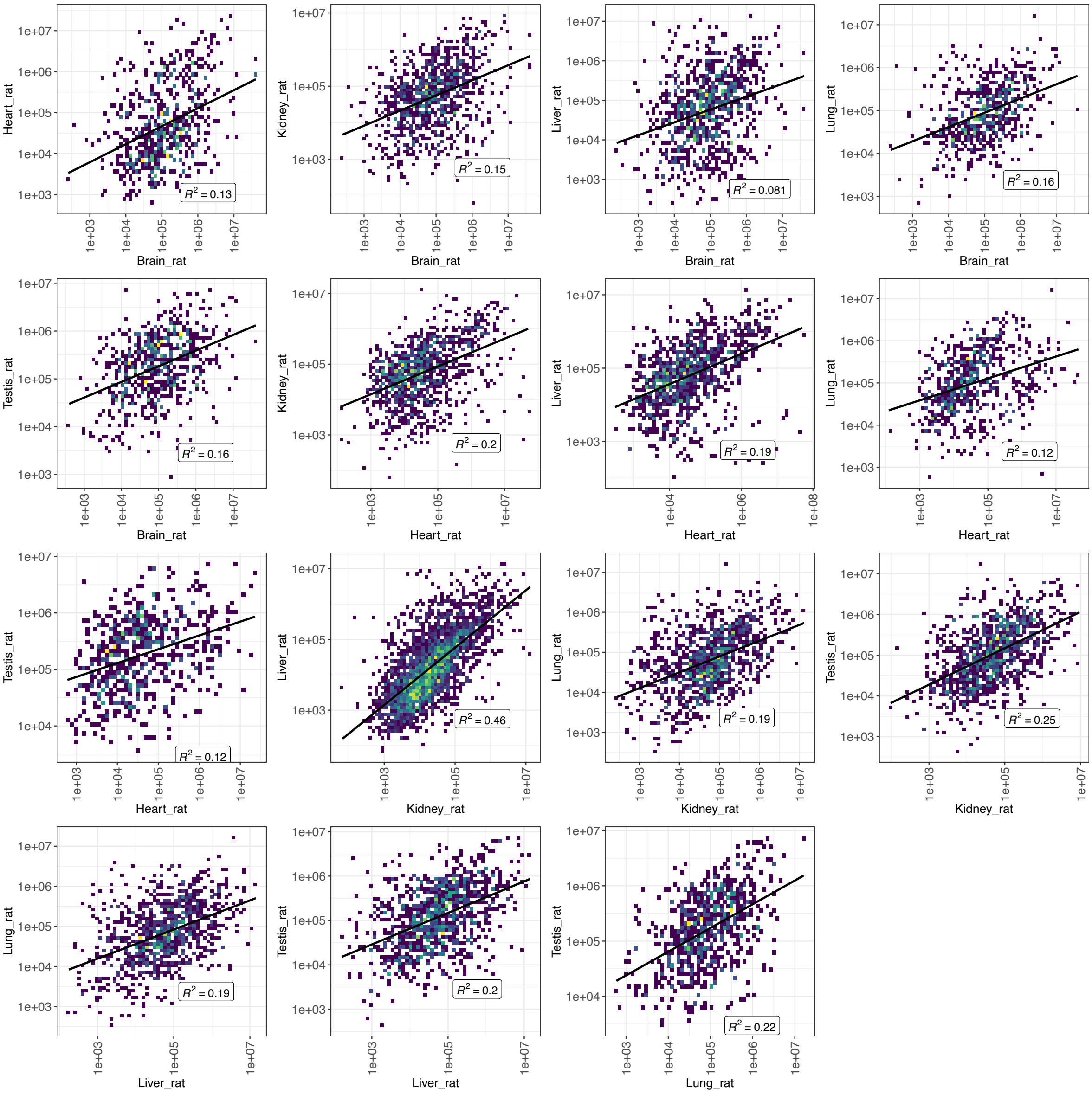

Supplementary Figure H. Correlation of protein expression between organs within rat.
