# Supplementary material for: Integrated view and comparative analysis of baseline protein expression in mouse and rat tissues
Source: PLoS Comput Biol. 2022 Jun 17;18(6):e1010174. doi: 10.1371/journal.pcbi.1010174 (PMC9246241; doi:10.1371/journal.pcbi.1010174)
Supplement: Attachment — Submitted filename: Response to reviewers for mouse_rat manuscript_JAV_28March.pdf [file pcbi.1010174.s010.pdf]

Response to reviewers

Integrated view and comparative analysis of baseline protein expression in mouse and rat tissues

We thank all reviewers for the time reviewing the manuscript. Please see our responses below.

Reviewer #1*:*

This manuscript integrated mouse and rat proteome datasets from published works and performed some correlation-based analysis across organs and species. I have the following questions regarding this manuscript and hopes the authors could address.

1. The paper seems to be a following up work from the group's previous paper (Jarnuczak, A.F., Najgebauer, H., Barzine, M. et al. An integrated landscape of protein expression in human cancer. Sci Data 8, 115 (2021). https://doi.org/10.1038/s41597-021-00890-2) but applies to mouse/rat proteomics data. The original work focused on human cell lines. The concepts and methodologies of the two papers are extremely similar. It would be nice if the authors could highlight the computational innovations introduced in the current paper not in the previous one.

Response: The overall proteomics data analysis methodology in both manuscripts is the same: label-free datasets are re-analysed and iBAQ expression values are provided as the resulting protein expression values. The main difference here when compared with the Jarnuczak *et al.* manuscript is that in that case the datasets were reanalysed in two large groups: those samples coming from cell lines and those samples coming from tumour tissues. We then realised that this strategy was not sustainable for re-analysing datasets at scale and integrating the results into a resource (Expression Atlas). Therefore, in this manuscript, each dataset was re-analysed separately. To make results more comparable and to try to remove batch effects, we performed the binning strategy for each dataset (which was not performed in the Jarnuczak *et al.* manuscript). This strategy also enabled the comparison of protein expression of orthologs across species (mouse, rat and human), which is performed in this manuscript for the first time (using the additional human expression data generated at: https://www.biorxiv.org/content/10.1101/2021.09.10.459811v2).

2. To my knowledge, PaxDB (https://pax-db.org/) is a very popular database for checking protein abundance. PaxDB contains many widely studied species including mouse and rat. It also has tissue information, protein interaction information and is regularly updated for years. To me, PaxDB seems to cover all values this paper could provide to me. It would nice that the authors could highlight the advantages of this paper that PaxDB doesn't have.

Response: PaxDB is a resource that relies on spectral counting data (plus downstream post-processing and normalisation) for reporting the quantitative protein expression values. This approach was quite popular in proteomics for a few years but nowadays, its use is limited and is no longer the state-of-the-art. One of the main reasons for this is that modern mass spectrometers have a setting called "dynamic exclusion". When operating instruments in this mode, the first scan measures the ions with the highest intensity (the most abundant ones). These masses are added to a temporary 'exclusion' list for a given period of time. Once the high intensity peaks have been sequenced and excluded the mass spectrometers can measure peaks under the threshold, thereby detecting less abundant peptides [1]. Dynamic exclusion gives the MS the ability to 'see' the less abundant ions, rather than repeatedly sequencing the same, abundant peptides. Since spectral counting relies on identified MS2 spectra (normally normalised considering the length of the protein), spectral counting can no longer be considered as a reliable proxy for peptide (and protein)

abundance. In any case, label-free intensity-based proteomics approaches are considered to be much more accurate than spectral counting and represent the current state-of-the-art.

However, to acknowledge the availability of protein expression values in PaxDB, we have now added a sentence in the introduction about this resource in the revised version of the manuscript. Additionally, in response to a query made by Reviewer 3, we have added a correlation analysis between the iBAQ data generated in this manuscript and the available data in PaxDB for different mouse organs. See all details in the response to Reviewer 3. The results of the analysis have been included in the revised version of the manuscript and as Supplementary material (Supplementary Figure S2).

3. One issue related to DDA based bottom-up proteomics is the reproducibility. One comparison that is missing in this paper is how well the proteins correlate with each other from different datasets for the same organ. This is critical. If the common proteins sampled from the same organ from two individual studies do not well correlate with each other, then to establish a global baseline for proteome across organs and species would be not very useful.

Response: Following the suggestions of the reviewer, we compared the FOT (Fraction Of Total) normalised iBAQ protein abundances (ppb) across datasets for the same organs to study their correlation in protein expression values. For mouse datasets we compared liver, heart and brain samples across multiple datasets. Supplementary Figure S1 (A to C) shows significant correlations of protein abundances of organs across datasets (liver: R^2 values from 0.59 to 0.74; heart: R^2 values ranging from 0.54 to 0.83; brain: R^2 values from 0.28 to 0.72).

For rat we could only perform the analysis on heart samples as this was the only organ that was shared between more than one dataset (PXD001839 and PXD013543). As shown in Supplementary Figure S1D a strong correlation (R^2=0.9) of protein expression was found between these two datasets. We have provided these comparison results as Supplementary file 4 in the revised version of the manuscript.

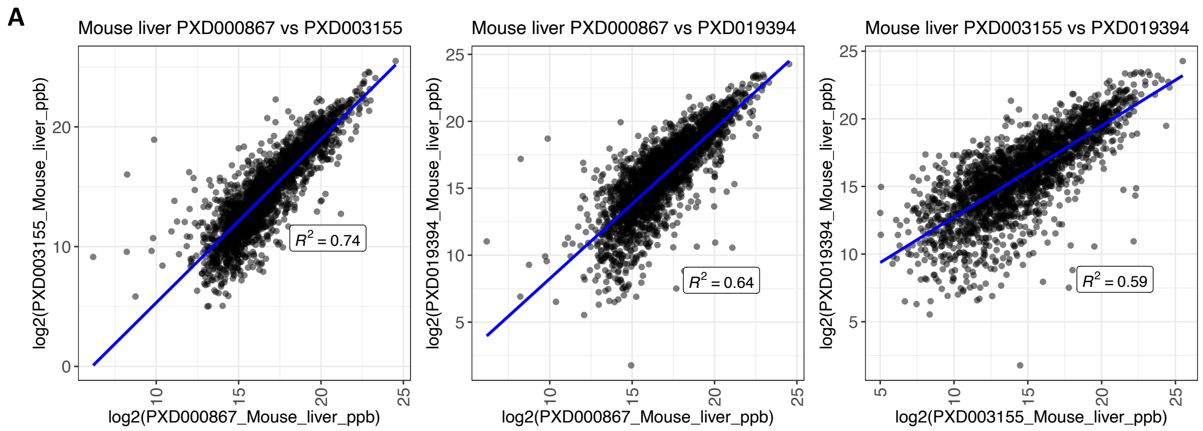

B

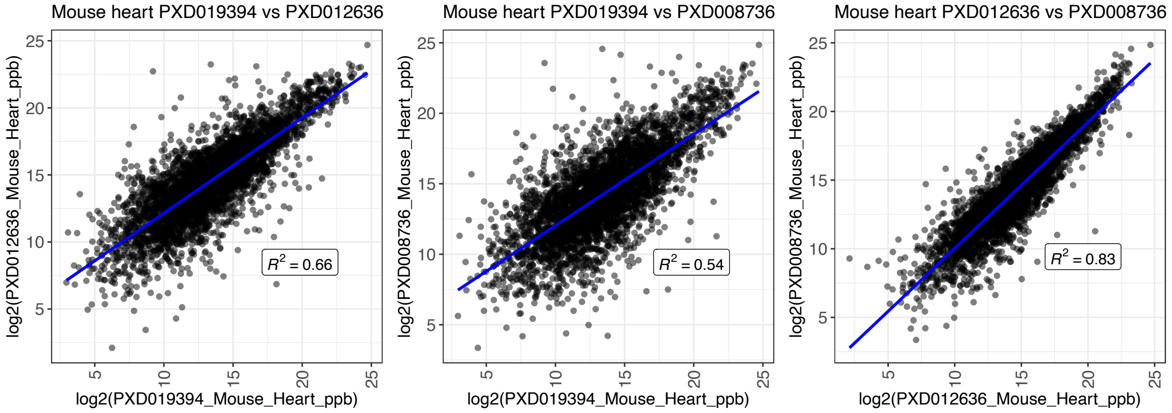
Mouse heart PXD019394 vs PXD012636
log2(PXD012636_Mouse_Heart_ppb)
log2(PXD019394_Mouse_Heart_ppb)
$R^2 = 0.66$
Mouse heart PXD019394 vs PXD008736
log2(PXD008736_Mouse_Heart_ppb)
log2(PXD019394_Mouse_Heart_ppb)
$R^2 = 0.54$
Mouse heart PXD012636 vs PXD008736
log2(PXD008736_Mouse_Heart_ppb)
log2(PXD012636_Mouse_Heart_ppb)
$R^2 = 0.83$

C

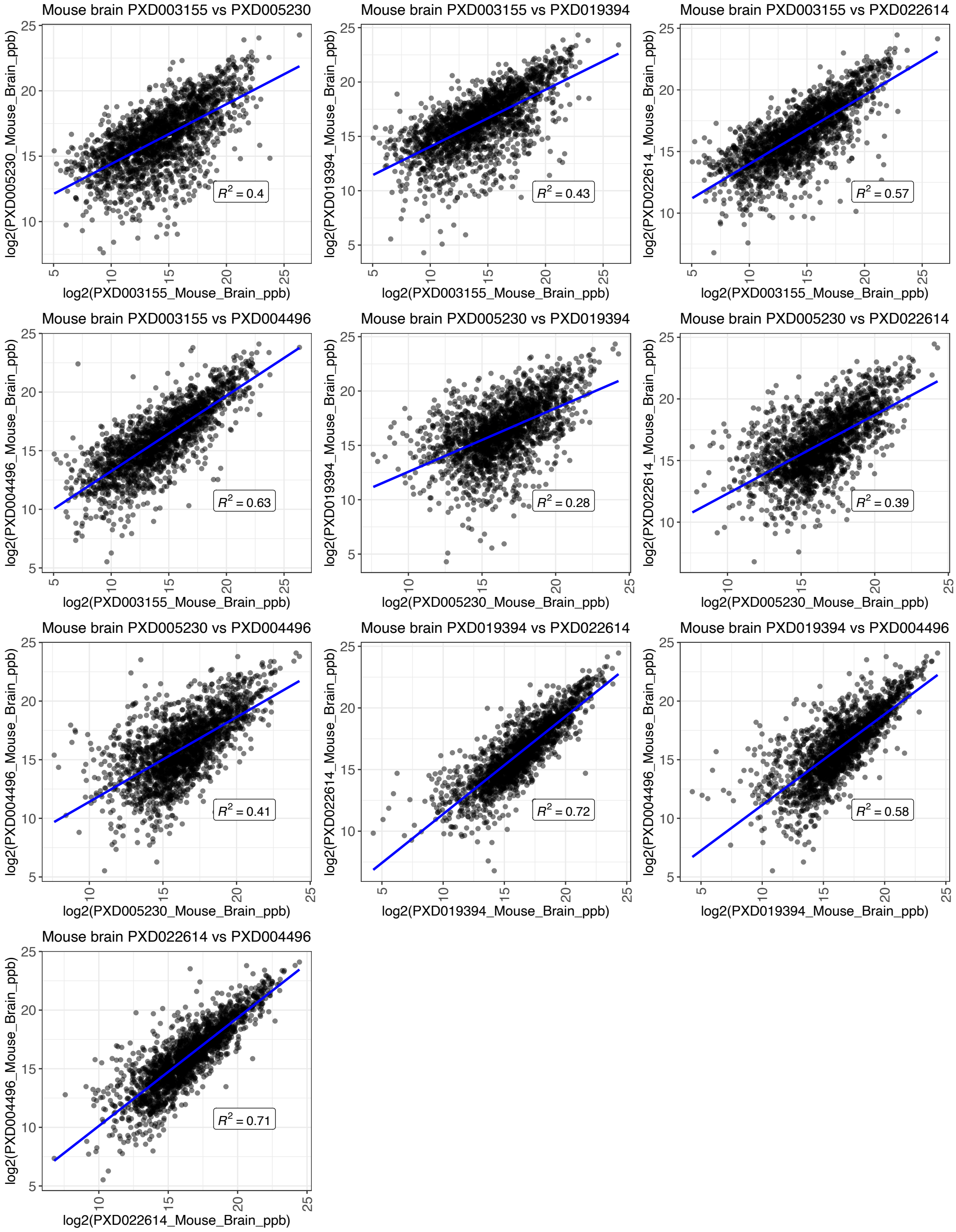
Mouse brain PXD003155 vs PXD005230
log2(PXD005230_Mouse_Brain_ppb)
log2(PXD003155_Mouse_Brain_ppb)
$R^2 = 0.4$
Mouse brain PXD003155 vs PXD019394
log2(PXD019394_Mouse_Brain_ppb)
log2(PXD003155_Mouse_Brain_ppb)
$R^2 = 0.43$
Mouse brain PXD003155 vs PXD022614
log2(PXD022614_Mouse_Brain_ppb)
log2(PXD003155_Mouse_Brain_ppb)
$R^2 = 0.57$
Mouse brain PXD003155 vs PXD004496
log2(PXD004496_Mouse_Brain_ppb)
log2(PXD003155_Mouse_Brain_ppb)
$R^2 = 0.63$
Mouse brain PXD005230 vs PXD019394
log2(PXD019394_Mouse_Brain_ppb)
log2(PXD005230_Mouse_Brain_ppb)
$R^2 = 0.28$
Mouse brain PXD005230 vs PXD022614
log2(PXD022614_Mouse_Brain_ppb)
log2(PXD005230_Mouse_Brain_ppb)
$R^2 = 0.39$
Mouse brain PXD005230 vs PXD004496
log2(PXD004496_Mouse_Brain_ppb)
log2(PXD005230_Mouse_Brain_ppb)
$R^2 = 0.41$
Mouse brain PXD019394 vs PXD022614
log2(PXD022614_Mouse_Brain_ppb)
log2(PXD019394_Mouse_Brain_ppb)
$R^2 = 0.72$
Mouse brain PXD019394 vs PXD004496
log2(PXD004496_Mouse_Brain_ppb)
log2(PXD019394_Mouse_Brain_ppb)
$R^2 = 0.58$
Mouse brain PXD022614 vs PXD004496
log2(PXD004496_Mouse_Brain_ppb)
log2(PXD022614_Mouse_Brain_ppb)
$R^2 = 0.71$

D

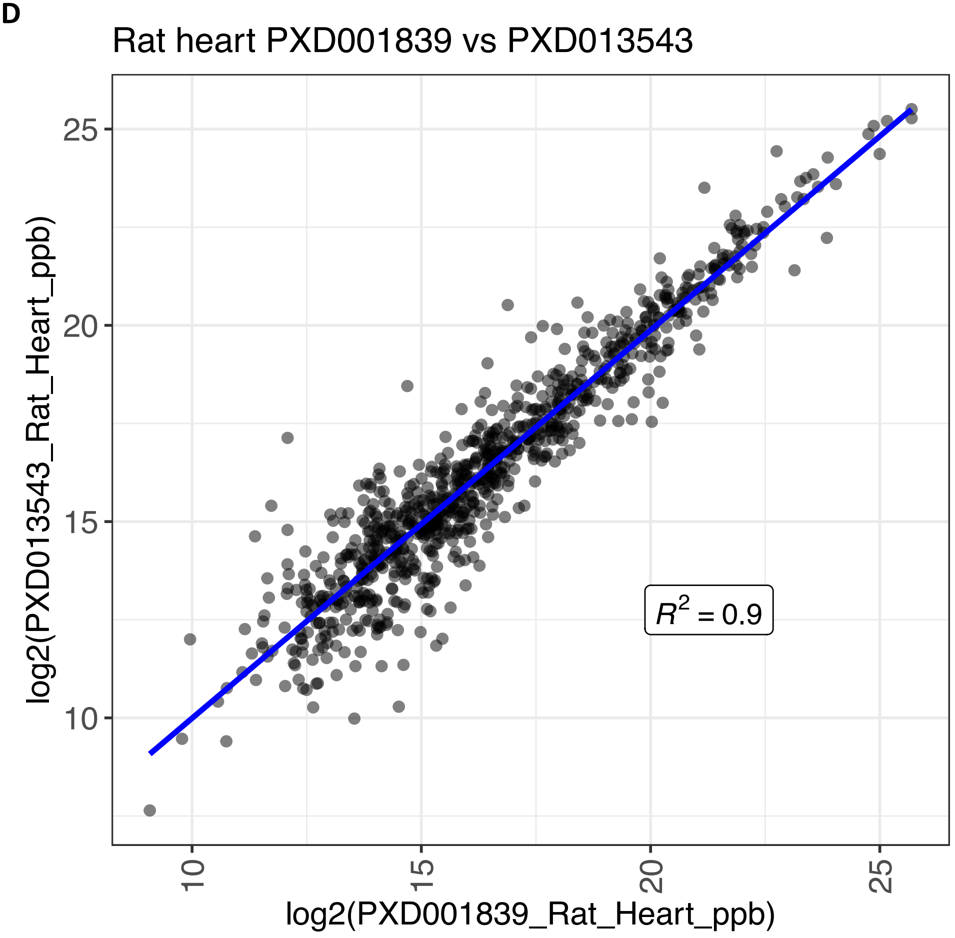

Figure S1. Correlation of protein expression values between datasets coming from (**A**) mouse liver, (**B**) mouse heart, (**C**) mouse brain and (**D**) rat heart.

Is deep fractionation proteome different from non-fractionation proteome? How do the authors adjust the difference using any kind of statistical modelling? For example, is TMT labelling different from iTRAQ labelling?

Response: In a proteomics experiment where fractionation is performed, the number of proteins identified (and quantified) increases significantly when compared to non-fractionated proteomes, because of the better separation of the peptides performed in the Liquid Chromatography step. Therefore, both types of studies (fractionated and non-fractionated) provide a different depth of the proteome. Direct comparability between both types of studies is therefore limited. In both cases, we performed the binning mechanism to increase comparability. In fractionated proteomes, the number of proteins included in the different bins were therefore much larger. But we think that the binning mechanism is equally suitable for both types of studies.

Out of the 23 datasets, samples in 12 of those datasets were fractionated (10 mouse datasets and 2 in rat). It is important to highlight that most studies in mouse were fractionated (10 out of 14 datasets) and most in rat were not (only 2 out of 9 datasets). This information has now been added

to Table 1. Finally, it should be mentioned that no TMT/iTRAQ datasets were used in this study, since they provide differential expression data (not baseline).

4. The title of this paper is about baseline expression of proteins. How did the baseline is set? By just the mean of expression or with any statistical justification?

Response: As explained in the manuscript, the baseline is set by including data only coming from tissues in “normal” conditions including control samples, in healthy state, generated without any perturbations.

5. How was the pathway analysis performed? If it was performed like gene set enrichment analysis, of course you will identify so many pathways with significant p-values. This will not provide many values. The interesting thing to see would be if particular pathways are detected in specific organs/tissue, but not in the others.

Response: For the pathway analysis, we first mapped all the canonical proteins from mouse and rat to the corresponding ortholog human proteins (this is needed for performing the analysis in Reactome). Then depending on the organ, we used the list of corresponding genes to perform the pathway analysis by directly searching against the Reactome Pathway Database. We performed an over-representation analysis, which could determine whether certain Reactome pathways were over-represented considering the input gene list for a given organ. Therefore, the pathways with significant p-values presented in Figure 8 were those where more proteins were expressed from those pathways than what would be expected by chance. From the pathway results, we did not specify pathways if they were particularly detected in specific organs/tissues. We provided here only a general view of the significant pathways from the organs of mouse and rat.

6. I am not super on top of the current mouse/rat proteomics literature. I am not sure if any targeted/DIA proteomics work has been done in mouse or rat. It would be nice to benchmark the DDA proteome to targeted/DIA proteome, since it was argued that targeted/DIA proteome measure is more accurate than DDA proteome.

Response: We agree with the reviewer in that this would be indeed be a good exercise to do, but we think this is out of the scope of this work. To the best of our knowledge, such comparison between DDA and DIA baseline results for the same mouse and rat tissue samples has not been performed so far (most of these studies have been performed so far in cell lines, e.g. [2]). If such study is not performed in the same samples, its value is very limited because in order to make a proper comparison across different methodologies (DDA and DIA), the participation of other variables should be avoided. Additionally, there are still very few suitable (re-usable) DIA datasets (if any at all) for baseline mouse tissue, and specially rat in the public domain. This means that this benchmarking at present cannot really be very comprehensive. Finally, the higher complexity of the analysis of DIA datasets (normally dependent on the availability of suitable spectral libraries) should not be underestimated. This would be a complete separate project on its own.

Reviewer #2:

Wang et al. described the results from a comparative analysis of publicly available rat and mouse proteomics data sets generated for fourteen tissues in the baseline (healthy) state. They verified that nearly half the detected proteins were significantly over-expressed in one or two types of tissue/organ system, and certain tissues such as tendon and testis have different sets of proteins dominating the abundance distribution given the uniqueness of their physiological and biological functions. They also noted that the protein expression levels are highly correlated between orthologs across species in line with general expectation. They are aware of the potential batch effects as individual data sets profile specific target organs only, and not all tissues and organs were analyzed in one single experiment. All in all, re-analyzing >20 proteomics datasets and standardizing the identification and quantification results (e.g. binning abundance levels) is a giant undertaking, and technical aspects of the work look solid in my opinion. Having said that, I believe the manuscript could have added more informative and exciting data analysis, rather than ending with the casual analysis of ortholog correlations (Figure 7) and generic functional enrichment-based clustering of organs (Figure 8).

Response: We thank the reviewer for their positive feedback.

Major recommendations

• *Expression Atlas has a large number of human proteomics data sets as well. One way to utilize the resource in the context of this paper would be to map unambiguous orthologs across the three species (as much as one can) in similar organs and tissues, and perform a projection analysis of proteins (e.g. t-SNE or UMAP) all at once. In such a visualization, for instance, serum amyloid A1 proteins should be almost uniquely synthesized in hepatocytes of the liver, and this protein from the three species should co-localize to the same proximal neighborhood in the projection plot (they can be labeled as SAA1_rat, SAA1_mouse, SAA1_human). It will be an interesting exercise to catalogue what proteins are quantitatively enriched in particular organ systems across the three species, and what proteins are not – the latter of which is no doubt the more intriguing part of the results. Describing the consistent and inconsistent findings across the species for endocrine (liver, pancreas, kidney, adrenal glands) and immune systems (spleen, if you have it) will be very useful for many investigators working on the molecular pathophysiology of a disease in specific organ system.*

Response: We agree this is a good exercise to undertake and have included a small section in the revised version of the manuscript outlying our use of UMAP and the methodology that we employed. We had available a large number of datasets generated from the previous human baseline proteome paper [3], where we also used the binning technique as shown in this manuscript (as also shown in the original section of the manuscript devoted to the analysis of orthologs across human, mouse and rat). This allowed us to compare gene orthologues across all 3 species, as recommended.

The UMAP plot showed strong localisation with regard to certain tissues such as heart, regardless of the species. Furthermore, when overlaying genes (corresponding to canonical proteins) on to the UMAP plot, we could see the specific genes localised to tissues (where they were known to be highly abundant within) and also that those genes were present across multiple species. We have included the results from this UMAP analysis in the revised version of the manuscript together with an extra figure (Figure 8). In addition, we have included the UMAP plot co-ordinates for each sample and additionally, the source data that can be overlaid on the plot, in the Supplementary File 9.

• I wonder if it is possible to acquire or assemble similar baseline mRNA expression data sets for matching sample types (MGI for mouse, RGD for rat, GTEx for human). This will allow you to evaluate tissue specific mRNA-protein ratio comparisons between species. While the lack of absolute quantification precludes the calculation of protein translation rates, comparison of pseudo-ratios of protein/mRNA across organ systems may turn out to be divergent across the species (or not).

Response: We thank the reviewer for this suggestion. As the reviewer says, it is indeed possible to do correlations between mRNA and protein expression values. However, there is the limitation that the samples are not the same ones, which of course limits the conclusions that can be extracted from these studies (as explained below for Reviewer 1 in the context of different methodologies).

There are multiple transcriptomics datasets available in the public domain. To address the reviewer's comment, we decided to perform a correlation with the overall baseline mRNA expression data from the resource MGI. The RNA-seq data from MGI provides expression values in TPMs in three categories, as 'Low', 'Medium' and 'High'. We converted these categorical values into the numeric values 1, 2 and 3, respectively.

In order to perform the comparison with the protein expression results included in this study, we re-binned the protein expression values obtained into 3 bins (originally, they were included in 5 different bins): 1, 2 and 3 to represent low, medium and high protein abundances. We then computed Spearman's correlation between paired organs of the two studies. We did not observe a strong correlation among organs. However, it is important to highlight that we performed a correlation with the aggregated expression values, as provided by MGI. Analogous correlation studies could be performed with each individual transcriptomic dataset separately, thus providing different results.

Mouse Organ	Spearman's_rho
Brain	0.222
Eye	0.297
Heart	0.346
Liver	0.459
Lung	0.229
Spleen	0.231
Testis	0.248

Unfortunately, we could not perform an analogous comparison for rat mRNA expression. In this case, it was not possible to bulk query/download overall RNA-seq expression of all rat genes from the resource RGD (like it is possible to do for MGI). As mentioned before, there are a number of suitable transcriptomics public datasets available in Expression Atlas that could be potentially used. Shortlisting several rat datasets based on their suitability and summarisation of gene expression over multiple datasets is a time-consuming process and given the limited time that we had to address the comments of the reviewers before resubmitting the manuscript, we decided not to perform this analysis, also because of the limited reach of their conclusions (again, the analysis were not performed in the same samples).

Minor comments

• Unlike tissue specific mRNA expression data sets (e.g. RNA-seq), MS/MS-based proteomics analyses

report identifiable, mostly soluble fraction of the proteome, which may differ by tissue types. For this reason, the current proportion-based normalization (ppb-iBAQ) may underestimate the missing fraction of the proteome in the denominator, i.e. the sum of all quantified proteins in that analysis of the tissue sample. In my humble opinion, the denominator should add a tissue-specific fudge factor to the sum, if one can estimate it. For instance, if you can find matching RNA-seq data sets, you can look at the overlap between identified proteins and the number of genes whose mRNA is expressed >1 in TPM in each tissue/organ type. This will reveal the fraction of identified and unidentified proteins, and you can add the estimate of the missing proteome abundance to the denominator. Of course this will require huge assumptions such as mRNA and protein levels are generally linearly correlated. I wonder whether this is a worthy investigation, or of interest to the authors. If you believe that the current normalization approach is robust enough and my suggestion is beyond the scope of the work, I will accept that.

Response: We thank the reviewer for this suggestion. We completely agree with the premise and the overall idea. One limitation would be the public availability of matching datasets, at least for mouse and rat. This is something that we could explore in a separate project, but we think it is outside the current scope of the work.

Reviewer #3:

The manuscript by Wang et al. describes a well-conducted and very relevant example of reuse of proteomics datasets available in public repositories. Twenty-three datasets from Pride corresponding to 211 samples originating from 34 tissues across 14 organs and including mouse and rat strains were used. First, they have elegantly extracted comparative protein expression maps between different tissues/organs of a given species to propose baseline protein expression profiles before deducing organ-specific enriched biological processes and pathways. The authors have previously applied an equivalent strategy on human baseline datasets coming from 32 different organs (Prakash A, et al. 2021, bioRxiv) and to compare human cell lines and tumour samples (Jarnuczak AF, et al. 2021, Sci Data). The originality of the present work relies in the cross-species comparisons that were further added. Indeed, the authors also conducted orthologs analyses to compare protein expression profiles of different tissue/organ types across mouse, rat and human samples. Finally, the output of the study has been integrated into the Expression Atlas, which is a nice way to make the work widely available.

Response: We thank the reviewer for their positive feedback.

As a specific remark, it is a shame that the current status of annotation/metadata availability of public datasets still requires, prior and fastidious, thorough manual cleaning/reannotation of the datasets before they can be reused for such a study. The authors should even more clearly highlight this shortcoming which constitutes a real brake to this type of studies and more generally to the re-use of public datasets.

Response: We agree with this statement. However, I think we have already highlighted enough this problem in the 'Discussion' section. It is really a difficult one to solve due to different reasons. We also highlight there some of our recent activities in that context, including the development of the MAGE-TAB-Proteomics format.

This work is worth being published in a journal like PLOS Computational Biology as this strategy can,

and should be, increasingly applied to a wide range of available –omics, and in particular, proteomics datasets.

I have only two major comments that should be adressed in a revised version of the manuscript :
- Instead of using finely extracted quantitative data from MaxQuant and then proceeding to a "coarse" binning, the authors should conduct the same analysis directly on spectral counting data (ex. length-normalised unique peptide counts). It would be very interesting to show whether/or not this has an impact on the results.

Response: Please see our response to Reviewer 1 related to spectral counting approaches and the resource PaxDB. We believe that spectral counting is no longer state-of-the-art in most proteomics settings for the reasons explained there, so we believe that making such comparison is not really very useful. However, we thought it would be interesting to compare our results with the spectral counting data generated by others. We have used then the values available in PaxDB for this comparison.

We compared our iBAQ normalised protein abundance results with the normalised protein abundances in PaxDB for mouse. We were only able to compare abundances from brain, heart, kidney, liver, lung, pancreas, and spleen as these were the common organs between PaxDB and our analysis. We saw generally a good correlation between these organs (Supplementary Figure S2). For rat however, we were unable to compare the abundances against PaxDB as there are no data there for individual organs (only available for the whole organism and several cell types).

In Supplementary Figure S2 it can be observed that the expression of low abundant proteins seems to be underestimated in PaxDB when compared with our iBAQ results, as shown by a S-shaped curve in the scatterplot in organs such as brain, heart, liver and lung. The 'dynamic exclusion' setting used by modern mass spectrometers allows to measure expression of low abundant proteins more accurately. This is a limitation when using spectral counting methods. We have included this figure in Supplementary File 4, and added a summary of our findings in the revised version of the manuscript.

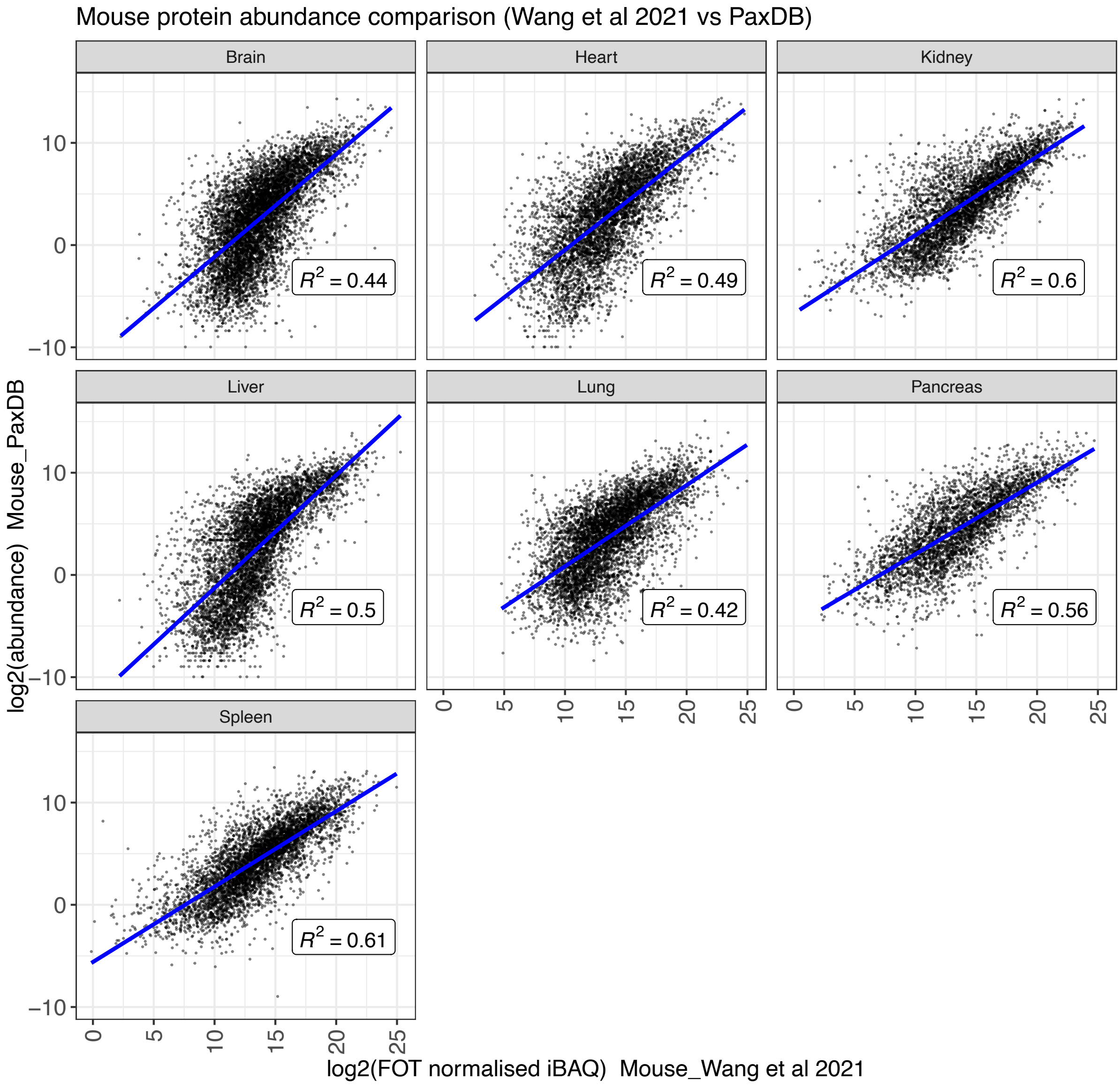

Figure S2. Comparison of mouse protein abundances in various organs between the results generated in study and the data available in PaxDB.

- The authors should correlate their results achieved with proteomics data with antibody-based data extracted from Protein Atlas. This later inclusion would provide an added-value to the work.

Response: The existing proteomics data in Human Protein Atlas (HPA) for mouse is available as protein-coding transcripts per million (pTPM), so data is derived from transcriptomics data, not from antibody-based data. Our understanding is that protein expression results for mouse proteins are therefore not available in the HPA. In this context, please see our response about the correlation between mRNA and protein expression values, performed as a response to one of the comments from Reviewer 2.

References

1. Hodge K. et al., Cleaning up the masses: exclusion lists to reduce contamination with HPLC-MS/MS. **J Proteomics**. 2013 Aug 2;88:92-103.

2. Fernandez-Costa C. et al., Impact of the Identification Strategy on the Reproducibility of the DDA and DIA Results. **J Proteome Res**. 2020 Aug 7;19(8):3153-3161.
3. Prakash A, García-Seisdedos D, Wang S, Kundu DJ, Collins A, George N, et al. An integrated view of baseline protein expression in human tissues. **bioRxiv**. 2021:2021.09.10.459811. doi: 10.1101/2021.09.10.459811.
