# Supplementary material for: Integrated view and comparative analysis of baseline protein expression in mouse and rat tissues
Source: PLoS Comput Biol. 2022 Jun 17;18(6):e1010174. doi: 10.1371/journal.pcbi.1010174 (PMC9246241; doi:10.1371/journal.pcbi.1010174)
Supplement: Attachment — Submitted filename: Response to reviewers for mouse_rat manuscript_JAV_30April.pdf [file pcbi.1010174.s011.pdf]

Response to reviewers Round 2

Integrated view and comparative analysis of baseline protein expression in mouse and rat tissues

We thank again all reviewers for their time reviewing the manuscript. Please see our responses below.

Reviewer #1:

Thank authors for addressing my questions. I still have questions regarding Question 2 and Question 5.

For Question 2, from my personal experience, spectral counts and ion intensity are highly correlated (also documented in many papers), there is not much difference in practice. Regarding PaxDb paper, I think they applied more interesting integration method to weigh each dataset and peptides and then benchmark using protein-protein interaction information. I think their work is quite innovative and interesting. It is hard to convince me this dataset has better quality or more useful than PaxDb.

Response: We are not stating that PaxDB is not a useful resource. But we think it cannot be ignored that the field as a whole has generally moved away from using spectral counting as the standard quantification method in most circumstances. We explained some of the reasons why in our previous rebuttal letter. We also included in the revised version of the manuscript Supplementary Figure 2, which contains a comparison between spectral counting and the iBAQ data generated in this study for mouse tissues.

For Question 5, I would suggest the authors to remove the pathway analysis section from the paper. The result is not very useful as detecting many enriched pathways is expected by the authors' setting, unless the organ/tissue specific pathways can be reported, which would be very interesting results.

Response: We respectfully disagree with the reviewer in this point and would like to keep the pathway analysis section in the manuscript. In fact, in Figure 9 it can be observed that some pathways are more enriched in certain organs, so these are in our view definitely interesting results.

I have no further questions.

Reviewer #2:

Authors have addressed my major concerns in the first review, and the manuscript has more substance than the initial submission. However, I strongly recommend the authors to consider rewriting the abstract, focusing on the key results rather than stating what was performed, e.g. comparison of orthologues and pathway enrichment analysis, etc.

Response: Following the suggestion of this reviewer, we have rewritten the abstract highlighting a bit more the reported results.
